# The use of healthcare contacts following a first diagnosis of chest pain among women with no obstructive coronary artery disease: results from the WOMANOCA nationwide cohort study

**DOI:** 10.1093/ehjqcco/qcaf051

**Published:** 2025-06-27

**Authors:** Jane Lange Dalsgaard, Michael Skov Hansen, Sofie Ronja Petersen, Lisette Okkels Jensen, Christian Backer Mogensen, Britt Borregaard

**Affiliations:** Department of Cardiology, University Hospital of Southern Denmark, Kresten Philipsens Vej 15, Aabenraa 6200, Denmark; Department of Regional Health Research, University of Southern Denmark, J.B. Winsløws Vej 19,3, Odense 5000, Denmark; Department of Clinical Research, University Hospital of Southern Denmark, Kresten Philipsens Vej 15, Aabenraa 6200, Denmark; OPEN, Open Patient Data Explorative Network, Odense University Hospital, J.B. Winsløws Vej 21,3, Odense 5000, Denmark; Department of Cardiology, University Hospital of Southern Denmark, Kresten Philipsens Vej 15, Aabenraa 6200, Denmark; Department of Clinical Research, University Hospital of Southern Denmark, Kresten Philipsens Vej 15, Aabenraa 6200, Denmark; Department of Clinical Research, University of Southern Denmark, J.B. Winsløws Vej 19,3, Odense 5000, Denmark; Department of Cardiology, Odense University Hospital, J.B. Winsløws Vej 4, Odense 5000, Denmark; Department of Regional Health Research, University of Southern Denmark, J.B. Winsløws Vej 19,3, Odense 5000, Denmark; Department of Clinical Research, University Hospital of Southern Denmark, Kresten Philipsens Vej 15, Aabenraa 6200, Denmark; Department of Emergency Medicine, Hospital of Southern Denmark, Kresten Philipsens Vej 15, Aabenraa 6200, Denmark; Department of Clinical Research, University of Southern Denmark, J.B. Winsløws Vej 19,3, Odense 5000, Denmark; Department of Cardiology, Odense University Hospital, J.B. Winsløws Vej 4, Odense 5000, Denmark

**Keywords:** Angina pectoris, Chest pain, Women’s health, Non-obstructive coronary artery disease, Healthcare utilization, Cohort studies

## Abstract

**Aims:**

Women with suspected angina but no obstructive coronary artery disease (CAD) may be at risk of frequent healthcare contacts due to persistent symptoms, but evidence is scarce. The study aimed to investigate healthcare use following a first diagnosis of angina or non-specific chest pain among a nationwide population of women with no obstructive CAD, compared to a reference population.

**Methods and results:**

The WOMANOCA cohort (*Wom*en with symptoms of *a*ngina or non-specific chest pain but *no* obstructive *CA*D) included all Danish women with a first diagnosis of angina or non-specific chest pain from 2009 to 2019 (following in registries for 3 years) and matched to asymptomatic reference women by age. Cox regression models assessed healthcare contacts between groups, reported as hazard ratios (HR) with 95% confidence intervals (CI). In total, 17 836 women were diagnosed with angina and 42 832 with non-specific chest pain, matched to 303 247 references. Women with angina and women with non-specific chest pain had a higher risk of all cardiac readmissions compared to the references (HR 3.24 95% CI 3.10–3.38 and HR 2.87 95% CI 2.78–2.97, respectively), with an increased association for angina compared to non-specific chest pain (HR 1.16 95% CI 1.10–1.22). Being a woman with non-specific chest pain was significantly associated with GP direct consultations, out-of-hours consultations, and ECGs compared to angina and the reference women.

**Conclusion:**

Despite no obstructive CAD, the WOMANOCA cohort had an increased use of new healthcare contacts, indicating a sustained healthcare burden among women with any type of chest pain.

## Structured graphical abstract

**Structured graphical abstract:**
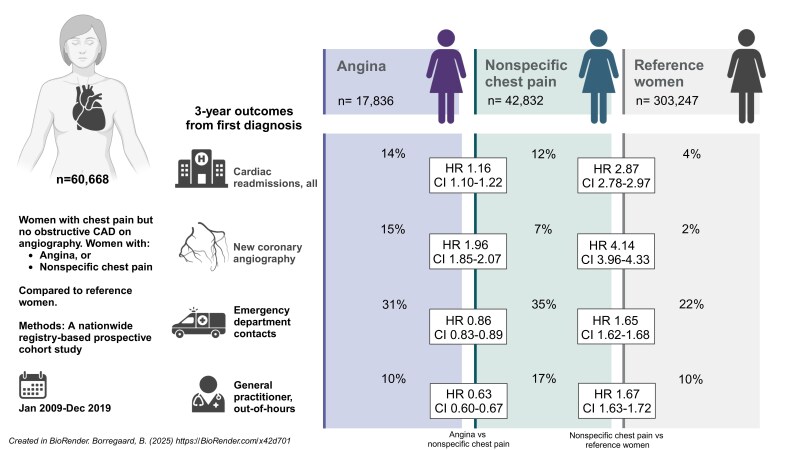

Key Learning Points What is already known:

- A substantial proportion of women with symptoms of angina have no obstructive coronary artery disease (CAD) on angiography.
- Angina/ischaemia with no obstructive coronary arteries (ANOCA/INOCA) is associated with a significant symptom burden and adverse clinical outcomes.
- There is limited population-level data on how women with non-obstructive CAD utilize healthcare services following their initial diagnosis.

What this study adds:

- This nationwide cohort study demonstrates that women with chest pain and no obstructive CAD have a higher rate of subsequent healthcare contacts compared to a background reference population of women, indicating an ongoing clinical burden.
- The findings challenge the perception that non-obstructive CAD is a benign condition and highlight unmet clinical needs in this population.
- The study underscores the importance of improving diagnostic pathways, follow-up strategies, and recognition of chest pain in women with no obstructive CAD to ensure appropriate management and resource allocation in clinical care.

## Introduction

Among women with chest pain, invasive coronary angiography (CAG) or non-invasive computed tomography coronary angiography (CTCA) are the cornerstones in diagnosing morphological changes with coronary obstructions and further treating symptoms.^1^ Even though the symptoms appear indicative of myocardial ischaemia,^2^ it is widely acknowledged that women often present with epicardial coronary stenosis <50%, meaning that they have no obstructive coronary artery disease (CAD) explaining their symptoms.^3^ As a result, invasive or non-invasive angiography often leads to a diagnosis of ‘no obstructive CAD’ or, in some cases, microvascular angina or angina/ischaemia with no obstructive coronary arteries (ANOCA/INOCA), and/or coronary spasm,^4^ typically preceded by a non-invasive stress test.

Accurately diagnosing women with chest pain and no obstructive CAD remains challenging, even in specialized cardiac centres. Differentiating between symptoms of chest pain caused by angina (presumed cardiac cause) and more non-specific chest pain due to non-cardiac causes is crucial, especially since diagnoses related to angina are often overlooked, with symptoms frequently mistaken for non-cardiac issues.^5^ Therefore, recent guidelines emphasize the need to tailor treatment based on symptoms,^6,7^ including a structured treatment approach to support patients with chest pain. Studies have shown that women with chest pain without obstructive CAD may experience reduced quality of life^8,9^ and increased morbidity.^4,10^ Similarly, our previous research confirms that self-reported health, including quality of life and mental health, is reduced regardless of whether symptoms of chest pain are due to angina or non-specific chest pain.^8^

The clinical trajectory post-discharge differs between the two groups of chest pain, women with symptoms of angina or symptoms of non-specific chest pain.^8^ Women with angina tend to require repeated procedures for obstructive CAD,^8,11^ leading to significant healthcare costs.^12,13^ We hypothesize that symptomatic women consume more healthcare services than the general female population, highlighting the need for a more structured approach. But, although significant healthcare utilization has been suggested, more extensive studies are needed.^4^

Thus, the objective of this study was to (i) describe differences in healthcare contacts among a nationwide population of Danish women with symptoms of angina or non-specific chest pain but no obstructive CAD compared to a background reference population of women and (ii) investigate the association between the groups and healthcare contacts within 3 years of follow-up following the first diagnosis.

## Methods

### Study design and population

This nationwide registry-based prospective cohort study of *wom*en with symptoms of *a*ngina or non-specific chest pain but *no* obstructive *CA*D on angiography (the WOMANOCA cohort), included adult Danish women with chest pain but no obstructive coronary artery disease on angiography. The women were admitted to the hospital for diagnostic evaluation due to angina-like symptoms between 1st January 2009 and 31st December 2019, with a 3-year follow-up. Reporting of the study adheres to the guidelines for strengthening the reporting of epidemiological observational studies (STROBE).^14^

### Setting and data sources

Data was obtained from Danish nationwide administrative and health registries.^15,16^ In Denmark, public hospitals are governed by five regions, supported by the universal tax-supported healthcare system. In addition, general practitioners (GPs) collaborate with all public hospitals and the 98 municipalities, providing community support.

The WOMANOCA population was identified by their unique personal identification number stored in the Danish Civil Registration System^15^ and linked to a unique hospital admission with a first-ever diagnostic code registered on symptoms of angina or non-specific chest pain.

Data was obtained from the following registries:

- The Danish National Patient Register (DNPR) [hospital encounters and procedures, using the International Classification of Diseases, 10th Revision (ICD-10)],^17^
- The Danish Civil Registration System^18^ (vital status: alive or date of death),
- The Danish Health Service Register^19^ (GPs and out-of-hour services, civil status, and ancestry),
- The Danish National Prescription Register^20^ (medication prescriptions),
- The Danish Education Register^21^ (educational level).

Data on cardiac risk factors and diagnostic invasive or non-invasive cardiovascular procedures, including results, was obtained from the nationwide Danish Heart Register (DHR).^22^ Danish Heart Register was established in 2010, and registration of diagnostic evaluation of CAG or CTCA is mandatory.

### The WOMANOCA study population

From the DNPR and DHR, we identified all adult Danish women (18 years or older) discharged from a public hospital with a first-ever primary or secondary ICD-10 diagnostic code corresponding to any symptoms of chest pain, divided into symptoms of angina or non-specific chest pain during the specified period 1st January 1st to 31st December 2019, with a 3-year follow-up. The follow-up period was determined based on data availability from Statistics Denmark. Data were retrieved in 2023, with the latest available follow-up extending to the end of 2022. Consequently, for women diagnosed with angina in 2019, follow-up ended in 2022, resulting in a maximum follow-up time of 3 years for the most recently enrolled patients. As an inclusion criterion, women had to undergo diagnostic evaluation from a clinically indicated invasive CAG or non-invasive CTCA during the same hospital contact.

**Figure 1:**
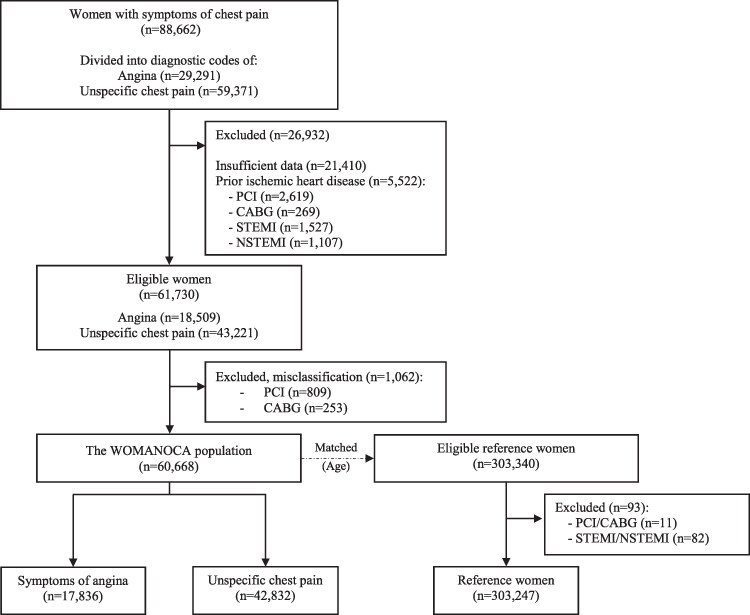
Flow diagram of the study population (women with symptoms of angina or non-specific chest pain) and the matched reference women.

To ensure the exclusion of women with a previous diagnosis of obstructive CAD, we excluded those who had undergone percutaneous coronary intervention (PCI), coronary artery bypass grafting (CABG), or had experienced an acute coronary syndrome (ACS) (*Figure 1*). Furthermore, we excluded those with insufficient data (*Figure 1*). The term ‘no obstructive CAD at angiography’ designates atheromatous without significant stenosis, angiographically normal coronary arteries, angiographically diffuse non-obstructive coronary arteries, and unspecified non-obstructive coronary arteries as one due to how it is categorized in the DHR emphasizing <50% stenosis in any epicardial coronary artery, which is the most commonly used cut-off point.^23^ Lastly, to prevent misclassification, we excluded patients who had undergone a PCI or CABG procedure within 90 days after the first CAG/CTCA.

The WOMANOCA cohort was stratified into two diagnostic subgroups based on clinical expert knowledge and clinical coding practice, as previously conducted^8^:

- Symptoms of angina: I20.0 (unstable angina), I20.1 (angina pectoris with documented spasm), I20.8 (other forms of angina pectoris), I20.8D (microvascular angina pectoris), I20.8E (stable angina pectoris), I20.9 (non-specific angina pectoris), and Z03.5C (observation for suspected stable angina pectoris).
- Non-specific symptoms of chest pain: Z03.4 (observations for suspected myocardial infarction), R07.3 (other chest pain), R07.4 (non-specific chest pain), and Z03.5 (observation for other suspected cardiovascular diseases).

The index date was defined as the hospital contact recording the first-ever diagnostic code corresponding to angina or non-specific chest pain and the diagnostic invasive CAG or non-invasive CTCA (and the matching date for reference individuals).

### Reference population

Each woman from the combined WOMANOCA population was matched with five reference women (1:5). The reference women was randomly drawn based on their identification number from the total Danish population in the Danish Civil Registration System and matched on age.

The matching criteria included the following: (i) the woman had to be a Danish resident and alive at the time of matching (although each individual may die or emigrate after being matched), (ii) the woman could not have any records of angina, non-specific chest pain or ACS, and (iii) the woman could not have any records of PCI or CABG, which would indicate a previous diagnosis of obstructive CAD. We allowed the reference women to change exposure status within the observation period.

### Baseline covariates

#### Demographic and clinical characteristics

Demographic data included age, cohabiting status (married or unmarried couple living together or multiple families living together), resident children, education level, and ethnicity (Danish, Western, and non-Western).

Cardiac risk factors included body mass index (BMI), smoking status, and diabetes (insulin or oral treatment, combined). Further, prescription medication within 90 days before admission was included, in addition to length of hospital stay, type of admission, type of angiography, procedural results, and Charlson comorbidity index scores (CCI)^24^ calculated based on ICD-10 primary codes within the past 10 years from index, grouped into none, one, two, or, greater than three comorbidities.^25,26^ International Classification of Diseases, 10th Revision codes, procedures, and medications are provided in Supplementary material online, *Table S1* and diagnostic code distribution in *Table S2*.

### Healthcare contacts within 3 years

Outcome data on healthcare contacts within 3 years after index admission included cardiovascular rehospitalizations i.e. hospitalizations due to cardiac diseases after the primary discharge ICD-10 diagnosis, all-cause hospitalizations, repeated invasive/non-invasive procedures (CAG/CTCA), revascularization (PCI/CABG), emergency department (ED) contacts (defined by Statistic Denmark as an acute hospital contact <12 h), GP consultation (direct contact either a physical-, video-, or telephone consultation), electrocardiogram (ECG) at GP (a presumed proxy for cardiovascular disease), and GP out-of-hours consultation (direct contact either a physical-, video-, or telephone consultation, or home visit).

### Statistical methods

Baseline characteristics were reported as percentages and numbers for categorical variables and mean [standard deviation (SD)] or median [interquartile range (IQR)] for continuous variables, as appropriate. The distribution of the variables was assessed graphically for normality. Descriptive statistics were used to describe the overall 3-year healthcare utilization among women with symptoms of angina or non-specific chest pain and the asymptomatic reference population.

The time to the first event was also visualized through cumulative incidence functions due to potential competing risk by death. Long-term prognostic outcomes, including mortality, ACS, and heart failure, were analysed using the full follow-up period (2009–22) and visualized with cumulative incidence functions to account for competing risk by death.

Univariate and multivariable Cox proportional hazards models were performed to investigate the association between symptoms of angina or non-specific chest pain and healthcare contacts. We compared both diagnostic groups to the matched reference population, separately and to each other. The models were adjusted for age, BMI, and comorbidity.^1,27^ Results were presented as hazard ratios (HRs) with a 95% confidence interval (CIs). Days from the index until the first event of interest, death, or censoring were used as the underlying time scale. Due to the discrete count distribution, we used a quasi-Poisson regression on telephone consultations and direct consultations at GP with results presented as incidence rate ratios (IRRs) with 95% CIs to examine the ratio between the total number of events per person. As a subgroup analysis, the potential association between CAG vs. CTCA and prognostic outcomes was investigated with Cox proportional hazard models. Model control for the Cox models was assessed graphically to assess functionality and fit. The proportional hazard assumption was evaluated using the Schöenfeld residuals.

Aalen's Additive Model was used to determine whether a time-split Cox regression could be conducted with a time split according to the pattern of the cumulative coefficients. Martingale residuals were used to investigate whether linearity was fulfilled for the continuous independent variables. The outcomes were evaluated and interpreted separately to avoid type-I error inflation.

Sensitivity analyses with cluster robust standard errors at the individual level were conducted to assess the robustness of the confidence intervals, as there were, by design, some controls that changed exposure status within the observation period.

The significance level was set to 5%. Statistical analyses were performed using STATA 18.0 (StataCorp LLC, TX, USA).

### Ethics statement

The study was registered with the internal record of the Region of Southern Denmark following Article 30 of the EU General Data Protection Regulation (No. 21/60601). According to Danish legislation, register-based studies do not require ethical approval in Denmark.

## Results

### Study population and baseline characteristics

A total of 88 662 women were diagnosed with symptoms of any kind of chest pain during the study period. Of these, 60 668 were eligible for analysis: 17 836 with diagnostic codes related to angina and 42 832 to non-specific chest pain (*Figure 1* and Supplementary material online, *Table S2*). The matched reference population consisted of 303 247 women.

Women with symptoms of angina were generally older than those with non-specific chest pain and the references population, with median ages of 62 years (IQR 54–70), 59 years (IQR 50–67), and 60 years (IQR 51–68), respectively (*Table 1*). A similar proportion of women across all groups were cohabiting (∼60%), and educational levels were comparable, with a majority having completed basic school or higher education (*Table 1*).

**Table 1:** Demographic and clinical baseline characteristics among women with symptoms of angina and non-specific chest pain and the reference women

	Symptoms of angina *n* = 17 836	Non-specific chest pain *n* = 42 832	Reference women *n* = 303 247	*P*-value^a^
Demographics				
Age, median (IQR)	62 (54–70)	59 (50–67)	60 (51–68)	<0.001
Age, *n* (%)				
≤49	2481 **(14)**	9597 **(22)**	60 506 **(20)**	
50–59	4807 **(27)**	12 872 **(30)**	88 323 **(29)**	<0.001
≥60	10 548 **(59)**	20 363 **(48)**	154 418 **(51)**	
Cohabiting, *n* (%)	10 768 **(60)**	26 272 **(61)**	181 385 **(60)**	<0.001
Resident children, *n* (%)	3239 **(18)**	11 115 **(26)**	71 695 **(24)**	<0.001
Educational level, *n* (%)				
Basic school	6970 **(39)**	15 219 **(36)**	102 655 **(34)**	<0.001
Upper secondary school/training	4727 **(27)**	11 595 **(27)**	86 422 **(29)**	
Higher education	5641 **(32)**	14 947 **(35)**	106 246 **(35)**	
Unknown	124 **(1)**	195 **(1)**	1833 **(1)**	
Missing	374 **(2)**	876 **(2)**	6091 **(2)**	
Ethnicity, *n* (%)^b^				
Danish	15 764 **(88)**	37 677 **(88)**	275 869 **(91)**	<0.001
Western	587 **(3)**	1510 **(4)**	12 056 **(4)**	
Not Western	1484 **(8)**	3644 **(9)**	15 318 **(5)**	
Cardiac risk factors				
BMI, median (IQR)	26 (23–30)	26 (23–30)	N/A	<0.001
BMI ≥ 25, *n* (%)	11 590 **(65)**	26 485 **(62)**	N/A	<0.001
BMI ≥ 30, *n* (%)	6075 **(34)**	13 414 **(31)**	N/A	<0.001
Current smoker, *n* (%)	2705 **(15)**	6920 **(16)**	N/A	<0.001
Former smoker, *n* (%)	5559 **(31)**	12 398 **(29)**	N/A	<0.001
Diabetes (insulin/oral treatment), *n* (%)	2026 **(11)**	3529 **(8)**	N/A	<0.001
Missing	15 810 **(89)**	39 303 **(92)**		
Length of hospital stay, days, *n* (%)				
1	17 307 **(97)**	41 564 **(97)**	N/A	0.98
2	267 **(2)**	645 **(2)**		
≥3	262 **(2)**	621 **(1)**		
Admission, planned, *n* (%)	17 014 **(96)**	40 595 **(95)**	N/A	<0.001
Angiographic procedure, *n* (%)^c^				
CAG	8597 **(48)**	11 593 **(27)**	N/A	<0.001
CTCA	10 656 **(60)**	33 513 **(78)**		
Coronary angiographic findings				
CAG, *n* (%)				
Ateromatosis without significant stenosis	3760 **(44)**	3913 **(34)**	N/A	<0.001
Normal coronary arteries	4837 **(56)**	7680 **(66)**		
CTCA, *n* (%)				
Diffuse non-obstructive coronary arteries	2454 **(23)**	6170 **(18)**	N/A	
Unspecified non-obstructive coronary arteries	8202 **(77)**	27 343 **(82)**		<0.001
Medication pre admission***, *n* (%)^d^				
Analgesics, *n* (%)				
Analgesics, combined^e^	5057 **(28)**	12 008 **(29)**	59 483 **(20)**	<0.001
Opioids	1858 **(10)**	4036 **(9)**	18 440 **(6)**	<0.001
Antidepressants, *n* (%)				
Anxiolytics	2116 **(12)**	4893 **(11)**	23 473 **(8)**	<0.001
Antidepressants	2060 **(12)**	5114 **(12)**	26 807 **(9)**	<0.001
Antithrombotic	6311 **(35)**	10 340 **(24)**	14 736 **(5)**	<0.001
Lipid-lowering	6366 **(36)**	10 920 **(26)**	36 227 **(12)**	<0.001
Antianginal	4216 **(24)**	5831 **(14)**	396 **(0)**	<0.001
Antianginal/Antihypertensive, *n* (%)				
Beta-blocker therapy	3803 **(21)**	6749 **(16)**	15 054 **(5)**	<0.001
Calcium antagonists	2927 **(16)**	5434 **(13)**	23 019 **(8)**	<0.001
Charlson comorbidity index, *n* (%)				
0	14 662 **(82)**	35 143 **(82)**	250 159 **(87)**	
1	1593 **(9)**	3876 **(9)**	13 616 **(5)**	<0.001
2	1210 **(7)**	2897 **(7)**	19 766 **(7)**	
3+	323 **(2)**	796 **(2)**	4305 **(2)**	

^a^ *P*-value set at 0.05.

^b^Missing on ethnicity was 1–4%.

^c^The proportion of coronary angiography (CAG) and computed tomography coronary angiography (CTCA) exceeds 100% due to the overlap in angiographic procedures.

^d^Prescripted medication registered from 3 months and until 1 day before the date of admission recording the first-ever diagnostic code corresponding to angina or non-specific chest pain.

^e^Analgesics combined consists of non-steroidal anti-inflammatory drugs (NSAIDs) and analgesics (see Supplementary material online, *Table S1*).

***Registered from 3 months and until 1 day before admission.

Women with angina were more likely to undergo invasive coronary angiography (CAG) (48%) compared to those with non-specific chest pain (27%) by the end of the follow-up period. In contrast, more women with non-specific chest pain were evaluated with non-invasive computed tomography coronary angiography (CTCA) (78% vs. 60% among women with angina). The proportion of women with cardiac risk factors such as being overweight, smoking history, and diabetes were similarly among women with angina and those with non-specific chest pain. Women with angina had the highest prevalence of prescribed heart medicine, particularly when compared to the reference population (*Table 1*).

### Healthcare contacts within 3 years following the first diagnosis of chest pain

*Table 2* depicts differences in new healthcare contacts over 3 years among women with angina, non-specific chest pain, and the reference population. A higher proportion of women with angina and non-specific chest pain had more healthcare contacts across all outcomes, compared to the reference population, with women with angina having the highest proportion of most contacts overall, except for out-of-hours-consultation and ED contacts, where the group of women with non-specific chest pain had most contacts (proportional). Both groups experienced higher proportions of all-cause readmissions and GP contacts compared to the reference population (*Table 2* and Supplementary material online, *Table S3*).

**Table 2:** Healthcare contacts within 3 years after the first diagnosis of angina or non-specific chest pain, compared to reference women

	Symptoms of angina *n* = 17 836	Non-specific chest pain *n* = 42 832	Reference women *n* = 303 247
Readmissions			
Cardiac readmission			
All cardiac readmissions, planned and unplanned			
Readmission (% readmitted), *n* (%)	2536 (14)	4951 (12)	12 769 (4)
Time to first readmission, days, median (IQR)	182 (46–529)	228 (57–585)	470 (225–756)
≤30 days, *n* (%)	461 (3)	820 (2)	443 (0.1)
≤1 year, *n* (%)	1632 (10)	3028 (7)	5060 (2)
Unplanned/acute cardiac readmission			
Readmission (% readmitted), *n* (%)	1796 (10)	3760 (9)	10 547 (4)
Time to first readmission, days, median (IQR)	354 (102–673)	366 (102–699)	502 (246–780)
≤30 days, *n* (%)	231 (1)	466 (1)	314 (0.1)
≤1 year, *n* (%)	913 (5)	1861 (4)	3913 (1)
All-cause readmission			
All-cause readmissions, planned and unplanned			
Readmission (% readmitted), *n* (%)	4518 (25)	10 801 (25)	55 354 (18)
Time to first readmission, days, median (IQR)	362 (147–681)	368 (143–679)	419 (186–715)
≤30 days, *n* (%)	299 (2)	830 (2)	2702 (1)
≤1 year, *n* (%)	2270 (13)	5369 (13)	24 719 (8)
New procedure (after index admission)			
Coronary angiography (CAG)			
New CAG, *n* (%)	2636 (15)	3056 (7)	5419 (2)
Time to first new CAG, days, median (IQR)	76 (31–390)	81 (32–427)	519 (287–787)
≤30 days, *n* (%)	650 (4)	731 (2)	122 (<1)
≤1 year, *n* (%)	1955 (11)	2230 (5)	1817 (1)
Computed tomography coronary angiography (CTCA)			
New CTCA, *n* (%)	557 (3)	1491 (4)	6204 (2)
Time to first new CTCA, days, median (IQR)	457 (67–834)	401 (56–799)	557 (328–805)
≤1 year, *n* (%)	248 (1)	718 (2)	1835 (1)
Percutaneous coronary intervention (PCI)			
PCI, *n* (%)	414 (2)	281 (1)	1496 (1)
Time to first PCI, days, median (IQR)	164 (90–482)	284 (97–690)	506 (253–790)
≤1 year, *n* (%)	280 (2)	158 (<1)	543 (<1)
Coronary artery bypass grafting (CABG)			
CABG, *n* (%)	74 (<1)	51 (<1)	320 (<1)
Time to first CABG, days, median (IQR)	129 (49–314)	126 (45–467)	483 (243–775)
≤1 year, *n* (%)	59 (<1)	36 (<1)	121 (<1)
Emergency Department (ED) contact			
ED visits, *n* (%)	5454 (31)	15 036 (35)	67 313 (22)
≤1 year, *n* (%)	2646 (15)	7255 (17)	27 391 (9)
General practitioner (GP)			
Direct consultation, regular opening hours			
Direct consultation, *n* (%)	17 087 (96)	41 260 (96)	277 144 (91)
Time to first direct consultation, days, median (IQR)	31 (11–85)	31 (10–87)	78 (28–191)
ECG, *n* (%)	1033 (6)	3016 (7)	13 461 (4)
Telephone consultation, *n* (%)	16 058 (90)	38 487 (90)	244 456 (81)
Out-of-hours consultation, *n* (%)	1857 (10)	7080 (17)	29 510 (10)
Death			
Mortality, *n* (%)	1352 (8)	2122 (5)	27 249 (9)

**Figure 2:**
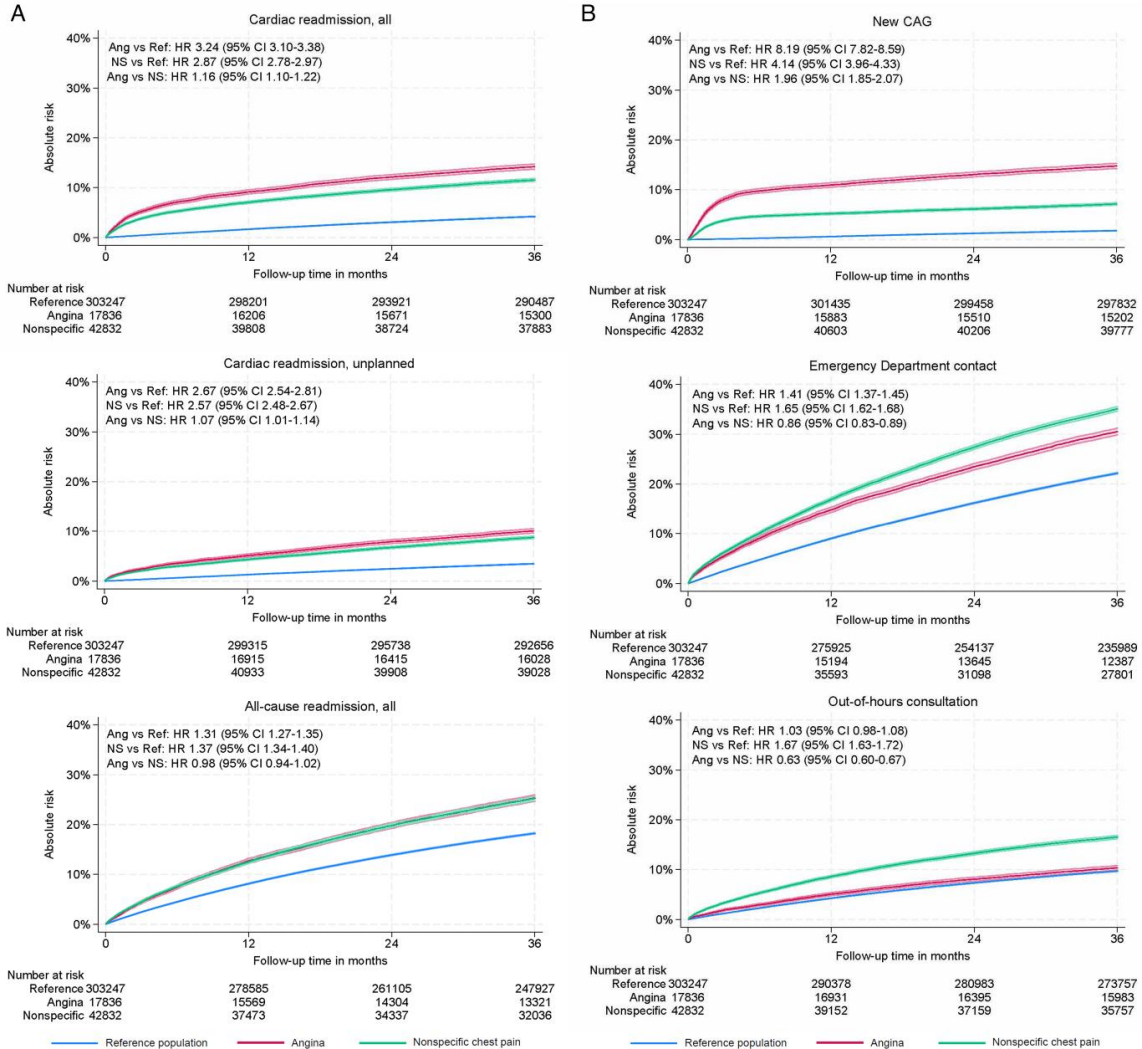
(*A* and *B*) Accumulated new events from the initial diagnosis up to 3 years of follow-up divided into cardiac readmission, all, cardiac readmission unplanned and all-cause readmission, all (*A*), and new coronary angiography, Emergency Department contact, out-of-hours consultation (*B*). Ang, angina; NS, non-specific; Ref, reference women. Cumulative incidence functions with a 95% confidence interval of the cumulative events within 3 years of follow-up. All survival curves include confidence intervals. All analyses were adjusted for Charlson comorbidity index. Analyses comparing angina vs. non-specific were adjusted for age, body mass index (BMI), and Charlson comorbidity index. Body mass index was only available for symptomatic women.

The most profound differences were seen in new CAGs. Women with angina had a higher proportion of CAGs (15%) compared to 7% in the non-specific chest pain group and 2% in the reference population. Also, women with angina had a higher proportion of all cardiac readmissions (14%) compared to non-specific chest pain (12%) and the reference population (4%). The proportion of unplanned cardiac readmissions was also higher in the angina group (10%) vs. non-specific chest pain (9%) and the reference population (4%). Contrary, women with non-specific chest pain had a higher proportion of out-of-hours consultations (17%) and emergency department contacts (35%) compared to women with angina (10% vs. 31%, respectively) and the reference population (10% vs. 22%, respectively) (*Table 2*). To distinguish between acute and planned healthcare utilization, outcomes were categorized separately in *Figure 2A* and *B* and in Supplementary material online, *Figure S1A*–*C*. A barchart illustrating the proportion of women experiencing the first event is provided in Supplementary material online, *Figure S2*.

### Characteristics associated with healthcare contacts within 3 years

*Figure 2A* and *B* also include characteristics associated with healthcare contacts between women with angina or non-specific chest pain, compared to the reference women, and between the two symptomatic groups. The analyses were adjusted for age, BMI, and co-morbidity. The adjusted and univariate analyses are provided in Supplementary material online, *Tables S4* and *S5*.

Women with angina had a higher risk of all (unplanned and planned) cardiac readmissions compared to the reference population (HR 3.24 95% CI 3.10–3.38). Similarly, also compared to the reference population, women with non-specific chest pain experienced an increased risk of all cardiac readmissions (HR 2.87 95% CI 2.78–2.97). When investigating the association between the two symptomatic groups, angina vs. non-specific chest pain, the association remained increased among those with angina (HR 1.16 95% CI 1.10–1.22). Unplanned cardiac readmissions were more frequent in women with angina (HR 2.67 95% CI 2.54–2.81) compared to the reference population. Planned cardiac readmissions, particularly within the first 6 months, had higher hazard ratios than unplanned events (see Supplementary material online, *Table S6*). Although procedures like CAG and PCI are generally outpatient procedures in Denmark, these early planned admissions may reflect brief hospital stays for persistent symptoms or further diagnostic evaluation. This suggests that initial diagnosis of angina or non-specific chest pain may, in some cases, be provisional and subject to refinement during early follow-up. Additionally, women with angina had an eight-fold higher risk of a new CAG (HR 8.19 95% CI 7.82–8.59) and a four-fold higher risk of PCI (HR 4.25 95% CI 3.81–4.74) when compared to the reference population. Although reduced HRs, the association remained increased among women with angina compared to women with non-specific chest pain (*Figure 2A* and *2B*, Supplementary material online, *Figure S1B*). A time-split Cox regression revealed a remarkably higher risk of CAG and PCI within the first 6 months after the initial CAG/CTCA (see Supplementary material online, *Table S6*).

In contrast, ED contacts were more common in women with non-specific chest pain compared to the reference population (HR 1.65, 95% CI 1.62–1.68). And the investigation of angina vs. non-specific chest pain and ED contacts was significantly reduced for the angina group (HR 0.86 95% CI 0.83–0.89). Being a woman with non-specific chest pain was significantly associated with GP direct consultations, out-of-hours consultations, and ECG procedures compared to both the reference population and women with angina (*Figure 2B*, Supplementary material online, *Figure S1C*). Both groups experienced higher proportions of all-cause readmissions and GP contacts than the reference population (*Figure 2A*, Supplementary material online, *Figure S1A* and *C*). The sensitivity analyses demonstrated the robustness of standard errors regarding controls becoming cases in the study period.

### Long-term prognostic outcomes

**Figure 3:**
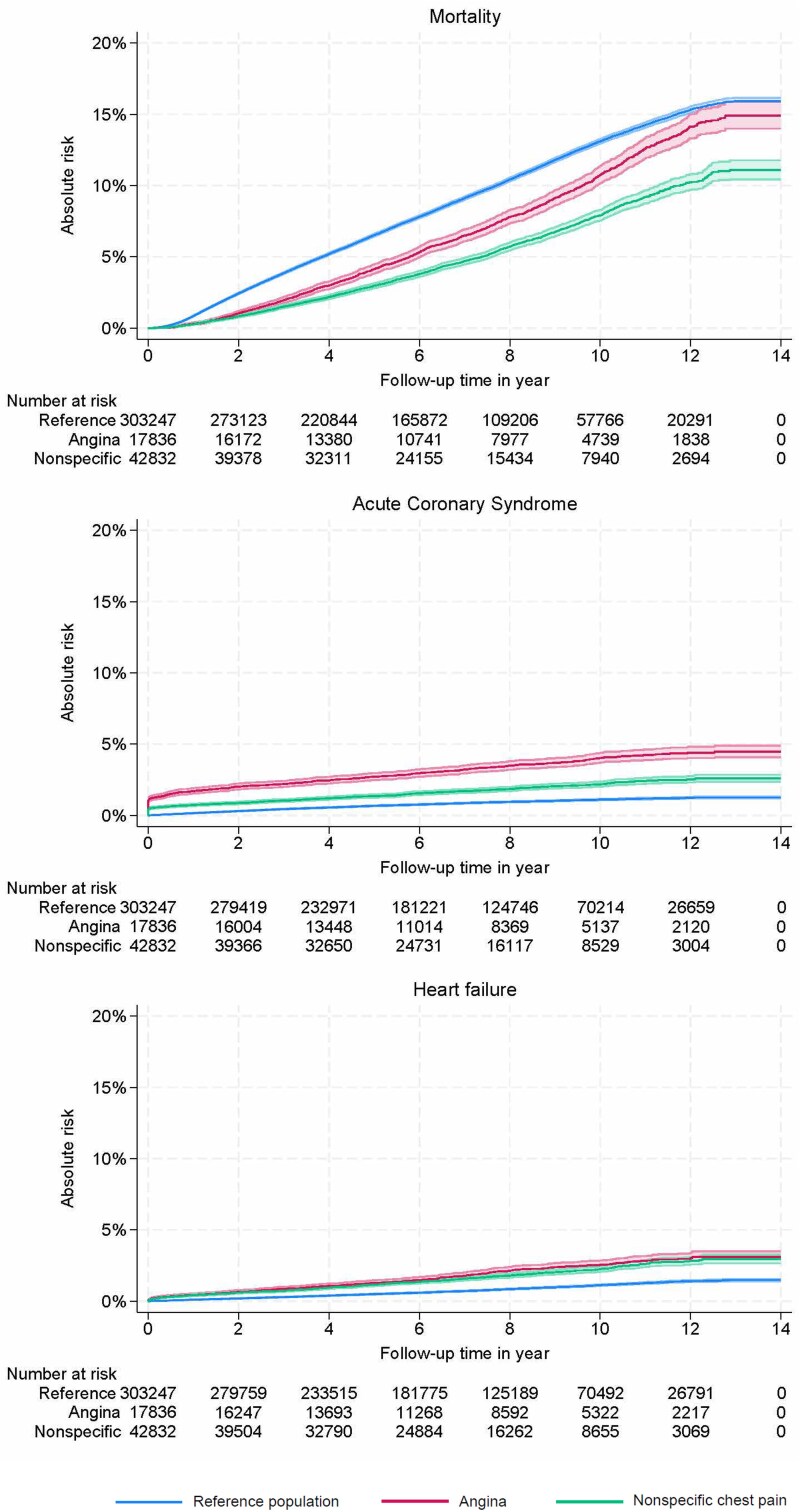
Accumulated long-term prognostic events from initial diagnosis to end of follow-up. Cumulative incidence functions with a 95% confidence interval, illustrating the occurrence of events over the full follow-up period (2009–22). The x-axis corresponds to the 14-year observation period from 2009 to 2022. All survival curves include confidence intervals.

The mean follow-up time was 7.55 years (SD 3.67) for women with angina, 6.92 years (SD 3.31) for those with non-specific chest pain, and 7.10 years (SD 3.43) for the reference population, with a follow-up range of 1–14 years across all groups. During the full follow-up period (2009–2022), the highest mortality rate was observed in the reference population (9%), followed by women with angina (8%) and those with non-specific chest pain (5%). In contrast, the incidence of acute coronary syndrome (ACS) was highest among women with non-specific chest pain (3%), compared to 2% in the angina group and 1% in the reference population. Similarly, the prevalence of heart failure was 2% in both the reference population and the non-specific chest pain group, while it was 1% in the angina group (*Figure 3*). The ICD-10 codes are provided in Supplementary material online, *Table S2*. On all prognostic outcomes, undergoing CAG vs. CTCA was associated with worse outcomes (mortality, ACS, and heart failure) among both women with angina and non-specific chest pain (see Supplementary material online, *Table S7*).

## Discussion

This is the first nationwide study to investigate the clinical trajectory among women discharged with a first-time diagnosis of angina or non-specific chest pain, but without obstructive CAD on angiography, compared to a general population of women. Women with symptoms of angina and those with non-specific chest pain were significantly associated with an increased use of new healthcare contacts within 3 years compared to the reference population, underscoring the persistent and troubling nature of their symptoms. Women with angina experienced a higher proportion of new cardiac-related healthcare contacts, including more CAGs and new revascularization, while women with non-specific chest pain had a higher proportion of ED contacts and out-of-hours contacts. In addition to a genuine disease progression, the difference may be due to ongoing cardiac concerns in women with angina. In contrast, distress and uncertainty might drive more ED visits among women with non-specific chest pain. This pattern highlights the potentially increased indecision among caring physicians as they evaluate and re-evaluate this subgroup of women. The lack of recognition of non-specific chest pain in women may be due to the ambiguity of their symptoms, which often do not align with typical cardiac pathology. As a result, women experiencing non-specific chest pain can be uncertain about the cause of their symptoms, leading to increased psychological distress and insecurity regarding their symptom aetiology. This, combined with the absence of a clear diagnosis, may drive more frequent visits to the emergency department. In contrast, women with angina are more likely to have an underlying cardiac condition, though it may not always be immediately recognized due to the absence of obstructive CAD. Consequently, these patients may seek emergency care less frequently, as their condition is more likely to be managed within the cardiac department.

Symptoms of angina among women vary widely, ranging from classic exertional chest pain to more atypical presentations, such as fatigue, shortness of breath, nausea, or dizziness.^5^ This variability is likely influenced by vasomotor dysfunction, including endothelial dysfunction, coronary artery spasm, and microvascular disease, which can cause fluctuations in symptoms over time.^6^ Such complexity may contribute to delayed recognition and differences in clinical presentation, further complicating diagnosis and management.

Given these challenges, both groups experienced higher proportions of all-cause readmissions and GP contacts compared with the reference population. The increased risk of new cardiac readmissions and CAGs among women with symptoms of angina but no obstructive CAD is in line with a previous research, including a Danish registry study of patients in the Eastern Denmark undergoing initial CAG due to stable angina. The study found an almost four-fold higher risk of recurrent hospitalization for angina vs. asymptomatic reference individuals.^11^ The current study, with a larger sample size and national coverage, confirms these findings 10 years later, highlighting the significant healthcare costs for women with symptoms of angina without obstructive CAD. This is consistent with other studies on angina hospitalization among women with symptoms of ischaemia but no obstructive CAD.^13,23,28^ Another interesting finding is the increased risk of new CAGs among the angina population within the first 6 months, often without accompanying revascularizations. This suggests that women with symptoms of angina without obstructive CAD undergo repeated and potentially unnecessary CAGs, indicating that the current diagnostic and therapeutic strategies may not fully address their symptoms, leading to ongoing medical consultations and interventions. However, due to our time-split analysis, some of these women may represent cases of initially underestimated or evolving obstructive CAD, suggesting that the initial testing failed to detect functionally significant disease, prompting re-evaluation.

Yet, our subgroup analyses reveal that women undergoing CAG have worse prognostic outcomes compared to those undergoing CTCA, including increased risk of death, ACS, and heart failure, highlighting the need for nuanced diagnostic approaches and tailored management strategies to improve long-term outcomes. Although women who underwent CAG showed worse long-term outcomes than those evaluated with CTCA, this difference may be influenced by confounding by indication. Those selected for invasive testing, such as CAG, likely had more severe symptoms or a higher baseline cardiovascular risk that was not fully accounted for in the adjusted variables. Thus, the differences in outcomes appear to stem from patient complexity rather than the diagnostic strategy used.

Although mortality was not the primary endpoint, women with angina and no obstructive CAD had lower observed mortality compared to the reference population. This aligns with the findings of Radico *et al.*^23^ indicating that symptoms of angina do not necessarily predict poorer outcomes. Recent studies provide further context for these findings. Berg *et al.*^29^ reported persistent impairments in HRQoL among women who experienced a myocardial infarction without obstructive coronary arteries (MINOCA), particularly physical and social functioning, highlighting the need for structured follow-up and support. In addition, Sterpetti *et al.*^30^ reported more favourable 1-year outcomes for women with myocardial ischaemia despite worse early procedural results. Together, these findings emphasize the need for a more nuanced understanding of outcomes in symptomatic women, highlighting the heterogeneity within this population and the need to distinguish prognostic subgroups.

Chest pain complaints, including non-specific chest pain, are among the most common reasons for visits to the emergency department,^2,7,31^ as reflected in our study comparing the two symptomatic groups to the reference population. Identifying patients without ‘typical/classic’ symptoms of obstructive CAD remains a challenge for emergency physicians, potentially leading to delayed and incorrect diagnosis and treatment.^5,7^ Some patients classified with non-anginal chest pain may in fact have had angina that was not recognized due to negative findings from additional investigations or misinterpretation of the clinical picture. This underscores the inherent challenge of relying on diagnostic coding in register-based studies, where large datasets provide valuable insights but may not always capture the full clinical reality. Such misclassification could contribute to the increased healthcare utilization observed in both symptomatic groups, as unrecognized angina may lead to persistent symptoms and long-term clinical outcomes. In particular, our findings suggest a potential under-recognition of ischaemic heart disease in the non-specific chest pain group, given their higher rates of ACS and heart failure compared to the reference population. This may indicate that traditional diagnostic pathways insufficiently detect non-obstructive ischaemic heart disease, such as microvascular dysfunction, in these women.

Contrary to our findings, a study by Ilangkovan *et al*.,^32^ including patients from Southern Denmark, concluded that the risk of cardiac-related events in patients referred to the emergency department with non-specific chest pain and no obstructive CAD had a prognosis similar to the general population. However, the study excluded patients suspected of CAD and who had an angiography performed during the index contact, making their patient population a more ‘low-risk’ group compared to the WOMANOCA cohort.

In general, current research on women with ‘angina suspected symptoms without obstructive CAD’ is difficult to compare due to varying definitions across studies.^2,23,33^ Our study included women with unstable angina diagnosed without myocardial infarction, a group often excluded in some studies. This inclusion, though, is consistent with other studies, which highlight that women are more likely to present with symptoms of non-obstructive CAD, including unstable symptoms, rather than only stable angina.^34^ Over the past two decades, studies have identified coronary microvascular dysfunction and epicardial vasospasm as key mechanisms of myocardial ischaemia in women. These conditions are frequently misdiagnosed, leading to recurrent angina, reduced quality of life, repeated hospitalizations, and unnecessary CAG.^8,35-37^ We speculate that due to diagnostic challenges and differences in treatment practices across hospitals, more women in our cohort might have these conditions, even if not indicated in their discharge diagnoses. This hypothesis is further supported by the higher use of antianginal medication among women in the angina group. Accurate diagnosis of these conditions remains challenging, with diagnostic techniques still evolving.^7,38^

In the WOMANOCA cohort, most women were substantially overweight, and nearly half had a history of smoking, reflecting a significant cardiac risk profile similar to other studies.^28,38^ Traditional cardiac risk factors, such as obesity (body mass index ≥30 kg/m^2^), smoking, hypertension, dyslipidaemia, diabetes, and mental stress, are well-established contributors to ischaemic heart disease in women.^38^ Obesity, particularly after menopause, also contributes to hypertension.^39^ Consequently, the higher use of lipid-lowering and antihypertensive medications in this cohort is likely related to the high prevalence of overweight women among cases. Worth highlighting, too, is the similar use of antidepressants across all groups, suggesting that the increased healthcare contacts are driven by physical symptoms rather than mental health issues. This is further supported by the higher use of analgesics and antianginal medications among these women compared to the reference population. The current study, corresponding to the latest European Guidelines,^35^ highlights the profound need to prevent long-term cardiovascular outcomes in these women. Finally, the increased healthcare utilization among women with chest pain but no obstructive CAD has significant economic implications. Frequent medical assessments, repeated procedures, and the need for ongoing symptom management contribute to higher healthcare costs. Addressing this issue through structured treatment approaches could help reduce these costs by improving symptom control and decreasing the need for repeated interventions. Findings from this study emphasize the importance of recognising and treating the symptoms of women with chest pain but no obstructive CAD. A multidisciplinary approach involving cardiologists, cardiac nurses, primary care physicians, and mental health professionals may be beneficial in supporting these patients.^40^ Clinicians should be aware of the potential for increased healthcare utilization and proactively develop individualized care plans addressing both physical and psychological aspects of the condition. Further research is needed to explore the underlying mechanisms of chest pain in women without obstructive CAD. Studies focusing on microvascular angina, endothelial dysfunction, and other potential causes are essential to develop targeted treatments. Additionally, research should investigate the long-term outcomes of different management strategies to identify the most effective approaches for improving quality of life and reducing healthcare utilization.

### Strengths and limitations

This study has notable strengths. The nationwide coverage enhances generalizability due to the large and diverse sample size of two symptom groups and the reference population. Utilizing highly valid Danish national health registries strengthens the representativeness and internal validity of the study. The registry-based cohort design minimizes selection bias by including baseline information on all eligible women and allows for life-long follow-up time. In addition, matching the nationwide cohort to a comparable background population by specific matching criteria provides a relevant benchmark.

However, there are limitations to consider. First, classifying ICD-10 codes into the two groups of symptomatic women involves a risk of misclassification bias, aligning with a recent validation study by the authors and the classification of diagnoses potentially differing across countries. Additionally, the non-anginal chest pain population may include individuals with angina whose condition was not recognized due to negative investigations or misinterpretation of the clinical picture by the treating physician. Second, excluding women who did not undergo angiography during the index contact may limit the applicability of the findings to all women with chest pain. Third, in 2016, the capital region of Denmark and the region of Zealand, accounting for slightly half of the Danish residents, implemented a new health platform to automatically extract real-time data from electronic medical records into the Danish Heart Register. Challenges with this extraction resulted in incomplete data, potentially leading to missing or insufficient data and a decreased likelihood of including all eligible symptomatic women in the cohort. Finally, variables on cardiac risk factors exist only in the angina and non-specific chest pain groups, leading to unmeasured confounders and potential measurement bias in the reference population.

## Conclusion

Compared to a cohort of matched reference women, women with symptoms of angina and women with non-specific chest pain had higher proportions of new healthcare contacts within 3 years after the first diagnosis. Being a woman with angina was associated with more cardiac-related contacts, while non-specific chest pain was associated with more general contacts. Despite the absence of obstructive coronary artery disease, the results highlight a need for structured follow-up among women with chest pain to prevent unnecessary health care contacts.

## Supplementary Material

qcaf051_Supplementary_Data

## Data Availability

The register-based data used in this study are derived from patient records and cannot be shared publicly due to confidentiality and privacy restrictions.
