# Supplementary material for: The use of healthcare contacts following a first diagnosis of chest pain among women with no obstructive coronary artery disease: results from the WOMANOCA nationwide cohort study
Source: Eur Heart J Qual Care Clin Outcomes. 2025 Jun 27;11(8):1396–408. doi: 10.1093/ehjqcco/qcaf051 (PMC12714392; doi:10.1093/ehjqcco/qcaf051)
Supplement: qcaf051_Supplementary_Data [file qcaf051_supplementary_data.zip › FigS1c_JLDA.pdf]

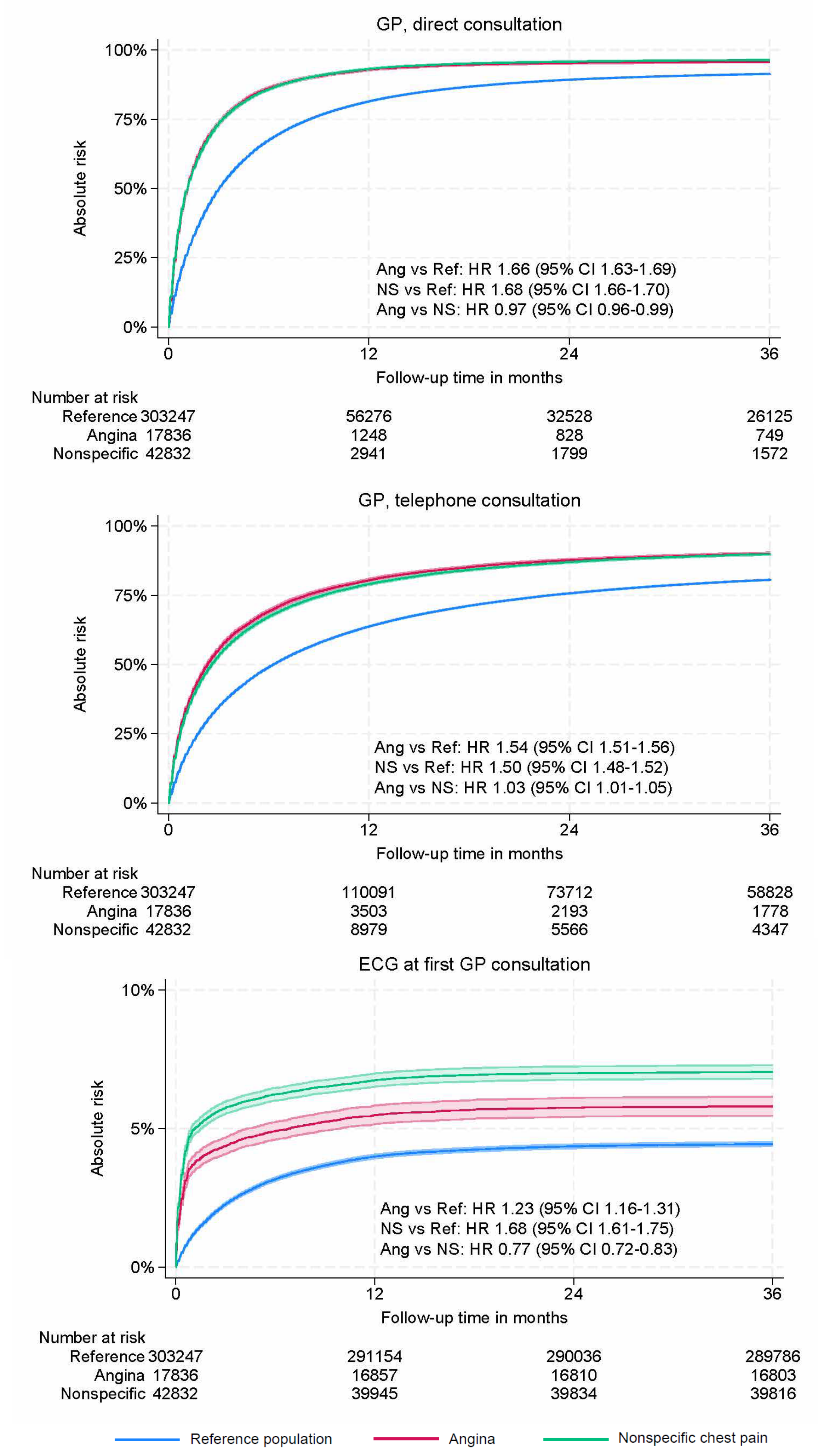
GP, direct consultation
Absolute risk
100%
75%
50%
25%
0%
0
12
24
36
Follow-up time in months
Ang vs Ref: HR 1.66 (95% CI 1.63-1.69)
NS vs Ref: HR 1.68 (95% CI 1.66-1.70)
Ang vs NS: HR 0.97 (95% CI 0.96-0.99)
Number at risk
Reference 303247 56276 32528 26125
Angina 17836 1248 828 749
Nonspecific 42832 2941 1799 1572
GP, telephone consultation
Absolute risk
100%
75%
50%
25%
0%
0
12
24
36
Follow-up time in months
Ang vs Ref: HR 1.54 (95% CI 1.51-1.56)
NS vs Ref: HR 1.50 (95% CI 1.48-1.52)
Ang vs NS: HR 1.03 (95% CI 1.01-1.05)
Number at risk
Reference 303247 110091 73712 58828
Angina 17836 3503 2193 1778
Nonspecific 42832 8979 5566 4347
ECG at first GP consultation
Absolute risk
10%
5%
0%
0
12
24
36
Follow-up time in months
Ang vs Ref: HR 1.23 (95% CI 1.16-1.31)
NS vs Ref: HR 1.68 (95% CI 1.61-1.75)
Ang vs NS: HR 0.77 (95% CI 0.72-0.83)
Number at risk
Reference 303247 291154 290036 289786
Angina 17836 16857 16810 16803
Nonspecific 42832 39945 39834 39816
Reference population
Angina
Nonspecific chest pain
