# Supplementary material for: The use of healthcare contacts following a first diagnosis of chest pain among women with no obstructive coronary artery disease: results from the WOMANOCA nationwide cohort study
Source: Eur Heart J Qual Care Clin Outcomes. 2025 Jun 27;11(8):1396–408. doi: 10.1093/ehjqcco/qcaf051 (PMC12714392; doi:10.1093/ehjqcco/qcaf051)
Supplement: qcaf051_Supplementary_Data [file qcaf051_supplementary_data.zip › Supplementary Material_JLDA.docx]

**Supplementary Material**

**Contents**

**Supplementary Table 1** ICD-10 diagnostic codes, procedure codes and ATC codes

**Supplementary Table 2** Distribution of diagnostic codes (angina and nonspecific chest pain)

**Supplementary Table 3** Healthcare contacts with recurrent events

**Supplementary Table 4** Characteristics associated with healthcare contacts (Adjusted)

**Supplementary Table 5** Characteristics associated with healthcare contacts (Unadjusted)

**Supplementary Table** **6** Characteristics associated with healthcare contacts; time-split Cox-regression

**Supplementary Table** **7** Angiographic procedure (CAG vs CTCA) associated with long-term prognostic outcomes

**Supplementary Figure 1a, 1b, and 1c** Accumulated new events from the initial diagnosis up to three years of follow-up.

**Supplementary Figure 2** Barchart demonstrating the proportion of women (angina, nonspecific chest pain, references) experiencing the first event within one month, one year, and after one year

**Table S1** ICD-10 diagnostic codes, procedure codes and ATC-codes

|  | **ICD-10 codes** | **Procedure codes** | **ATC codes** |
| --- | --- | --- | --- |
| **Procedure codes** | | | |
| Invasive CAG | N/A | UXAC85, UXAC85A, UXAC85B, UXAC85C | N/A |
| Non-invasive CTCA | N/A | UXCC00A | N/A |
| **Exclusion criteria codes** | | | |
| STEMI | I21.0B, I21.1B, I21.3, I21.3A, I21.3B, I21.3C, I21.9 | N/A | N/A |
| NSTEMI | I21.0A, I21.1A, I21.4 | N/A | N/A |
| PCI | N/A | KFNG, KFNF, KFNG00-KFNG05A | N/A |
| CABG | N/A | KFNA, KFNB, KFNC, KFND, KFNE, KFNH20 | N/A |
| **Long-term prognostic codes** | | | |
| ACS | STEMI and NSTEMI codes, including I20.0, I20.0B, I20.0C | N/A | N/A |
| Heart failure | I50.0, I50.0A, I50.1, I50.1A I50.1C, I50.1D, I50.9 | N/A | N/A |
| **Prescription medication** | | | |
| Analgesics combined | N/A | N/A | M01AE, M02AA, N02BA, N02BB, N02BE, N02BF, N02BG |
| Opioids | N/A | N/A | N02AA, N02AB, N02AC N02AD, N02AE, N02AF, N02AG, N02AJ, N02AX |
| Anxiolytics | N/A | N/A | N05A, N05B, N05C |
| Antidepressants | N/A | N/A | N06AA, N06AB, N06AX |
| Antithrombotic | N/A | N/A | B01AC04, B01AC06 |
| Lipid-lowering | N/A | N/A | C10AA, C10AB, C10AC, C10AD  C10AX09, C10AX12 |
| Antianginal | N/A | N/A | C01DA02, C01DA08, C01DA14, C01EB17  C01DX16 |
| Beta blocker therapy | N/A | N/A | C07AB |
| Calcium antagonists | N/A | N/A | C08CA, C08DA, C08DB |

N/A; Not applicable

**Table S2** The distribution of the diagnostic codes of angina and nonspecific chest pain

| **Diagnsotic codes* related to symptoms of angina (N=17,836)** |
| --- |
| Unstable angina, n=574 (3.2%) |
| Angina pectoris with documented spasm, n= 108 (0.6%) |
| Other forms of angina pectoris, n= 315 (1.8%) |
| Microvascular angina pectoris, n= 29 (0.2%) |
| Stable angina pectoris, n= 511 (2.9%) |
| Nonspecific angina pectoris, n= 9,382 (52.5%) |
| Observation for suspected stable angina pectoris, n= 6,917 (38.8%) |
| **Diagnostic codes** related to nonspecific symptoms of nonspecific chest pain (N=42,832)** |
| Observations for suspected myocardial infarction, n=5,265 (12.3%) |
| Other chest pain, n= 1,639 (3.8%) |
| Nonspecific chest pain, n=7,171 (16.7%) |
| Observation for other suspected cardiovascular diseases, n=28,757 (67.1%) |
| *94.2% are primary ICD-10 diagnsotic codes, and 5.8% are secondary ICD-10 diagnsotic codes.  **96.4% are primary ICD-10 diagnsotic codes, and 3.6% are secondary ICD-10 diagnotic codes. |

**Table S3:** Healthcare contacts within three years after the first diagnosis of angina or nonspecific chest pain, compared to reference women, including recurrent events

|  | **Angina**    **(n=17,836)** | **Nonspecific chest pain**  **(n= 42,832)** | **Reference women**    **(n=303,247)** |
| --- | --- | --- | --- |
| **Readmissions** |  |  |  |
| **Cardiac readmissions** |  |  |  |
| **All cardiac readmissions, planned and unplanned** | |  |  |
| Readmissions (% readmitted), n (%)  Time to first readmission, days, median (IQR)  ≤ 30 days, n (%)  ≤ 1 year, n (%)  > 1 year, n (%)  Number of readmissions, n (%)  0  1  2  ≥3 | 2,536 (14)  182 (46-529)  461 (3)  1,632 (10)  904 (5)  15,300 (86)  1,745 (10)  470 (3)  321 (2) | 4,951 (11)  228 (57-585)  820 (2)  3,028 (7)  1,923 (4)  37,881 (88)  3,440 (8)  910 (2)  601 (3) | 12,769 (4)  470 (225-756)  443 (0.1)  5,060 (2)  7,709 (3)  290,468 (96)  10,250 (3)  1,695 (1)  834 (0.3) |
| **Unplanned/acute cardiac readmission** |  |  |  |
| Readmissions (% readmitted), n (%)  Time to first readmission, days, median (IQR)  ≤ 30 days, n (%)  ≤ 1 year, n (%)  > 1 year, n (%)  Number of readmissions, n (%)  0  1  2  ≥3 | 1,796 (10)  354 (102-673)  231 (1)  913 (5)  883 (5)  16,040 (90)  1,330 (8)  295 (2)  171 (1) | 3,760 (9)  366 (102-699)  466 (1)  1,861 (4)  1,899 (4)  39,072 (91)  2,792 (7)  607 (1)  361 (1) | 10,547 (4)  502 (246-780)  314 (0.1)  3,913 (1)  6,634 (2)  292,700 (97)  8,902 (3)  1,199 (0.4)  446 (0.1) |
| **Planned cardiac readmission** |  |  |  |
| Readmissions (% readmitted), n (%)  Time to first readmission, days, median (IQR)  ≤ 30 days, n (%)  ≤ 1 year, n (%)  > 1 year, n (%)  Number of readmissions, n (%)  0  1  2  ≥3 | 1,095 (6)  180 (46-518)  182 (1)  711 (4)  384 (2)  16,741 (94)  904 (5)  144 (1)  47 (0.3) | 1,831 (4)  202 (61-552)  240 (1)  1,167 (3)  664 (2)  41,001 (96)  1,513 (4)  237 (1)  81 (0.2) | 3,149 (1)  521 (266-797)  69 (0.02)  1,079 (0.3)  2,070 (0.7)  300,098 (99)  2,712 (1)  349 (0.1)  88 (0.0) |
| **All-cause readmissions** |  |  |  |
| **All-cause readmissions, planned and unplanned** | |  |  |
| Readmissions (% readmitted), n (%)  Time to first readmission, days, median (IQR)  ≤ 30 days, n (%)  ≤ 1 year, n (%)  > 1 year, n (%)  Number of readmissions, n (%)  0  1  2  ≥3 | 4,518 (25)  362 (147-681)  299 (2)  2,270 (13)  2,248 (13)  13,318 (75)  2,750 (15)  928 (5)  840 (5) | 10,801 (25)  368 (143-679)  830 (2)  5,369 (13)  5,432 (13)  32,031 (75)  6,455 (15)  2,106 (5)  2,240 (5) | 55,354 (18)  419 (186-715)  2,702 (1)  24,719 (8)  30,635 (10)  247,893 (82)  34,469 (11)  10,542 (4)  10,343 (3) |
| **Unplanned/acute all-cause readmissions** |  |  |  |
| Readmissions (% readmitted), n (%)  Time to first readmission, days, median (IQR)  ≤ 30 days, n (%)  ≤ 1 year, n (%)  > 1 year, n (%)  Number of readmissions, n (%)  0  1  2  ≥3 | 2,993 (17)  477 (215-778)  153 (1)  1,217 (7)  1,776 (10)  14,843 (83)  1,981 (11)  543 (3)  469 (3) | 7,202 (17)  480 (223-778)  395 (1)  2,827 (7)  4,375 (10)  35,630 (83)  4,695 (11)  1,270 (3)  1,237 (3) | 36,865 (12)  504 (250-786)  1,114 (0.4)  13,394 (4)  23,471 (8)  266,382 (88)  24,582 (8)  6,526 (2)  7,757 (2) |
| **Planned all-cause readmissions** |  |  |  |
| Readmissions (% readmitted), n (%)  Time to first readmission, days, median (IQR)  ≤ 30 days, n (%)  ≤ 1 year, n (%)  > 1 year, n (%)  Number of readmissions, n (%)  0  1  2  ≥3 | 2,331 (13)  463 (207-773)  71 (0.4)  953 (5)  1,378 (8)  15,505 (87)  1,768 (10)  373 (2)  190 (1) | 5,750 (13)  473 (213-776)  237 (1)  2273 (6)  3477 (8)  37,082 (87)  4,220 (10)  1,001 (2)  529 (1) | 28,061 (9)  492 (237-782)  951 (0.3)  10,488 (3)  17,573 (6)  275,186 (91)  21,049 (7)  4,596 (2)  2,416 (1) |
| **New procedure (after index admission)** |  |  |  |
| **Coronary Angiography (CAG/CTCA)** |  |  |  |
| **Coronary angiography (CAG)** |  |  |  |
| New CAG, n (%)  Time to first new CAG, days, median (IQR)  ≤ 30 days, n (%)  ≤ 1 year, n (%)  > 1 year, n (%)  Number of new CAGs, n (%)  0  1  2  ≥3 | 2,636 (15)  76 (31-390)  650 (4)  1,955 (11)  681 (4)  15,200 (85)  2,231 (13)  295 (2)  110 (1) | 3,056 (7)  81 (32-427)  731 (2)  2,230 (5)  826 (2)  39,776 (93)  2,675 (6)  314 (1)  67 (0.2) | 5,419 (2)  519 (287-787)  122 (0.04)  1,817 (1)  3,602 (1)  297,828 (98)  4,727 (2)  575 (0.2)  117 (0.0) |
| **Computed tomography coronary angiography (CTCA)** | |  |  |
| New CTCA, n (%)  Time to first new CTCA, days, median (IQR)  ≤ 30 days, n (%)  ≤ 1 year, n (%)  > 1 year, n (%)  Number of new CTCAs, n (%)  0  1  2  ≥3 | 557 (3)  457 (67-834)  93 (1)  248 (2)  309 (2)  17,279 (97)  412 (2)  135 (1)  10 (0.1) | 1,491 (4)  401 (56-799)  252 (1)  718 (2)  773 (2)  41,341 (97)  1,237 (3)  230 (1)  24 (0.1) | 6,204 (2)  557 (328-805)  74 (0.02)  1,835 (1)  4,369 (1)  297,043 (98)  5,214 (2)  935 (0.3)  55 (0.0) |
| **Revascularisation** |  |  |  |
| **Percutaneous coronary intervention (PCI)** |  |  |  |
| PCI, n (%)  Time to first PCI, days, median (IQR)  ≤ 1 year, n (%)  > 1 year, n (%)  Number of PCI, n (%)  0  1  2  ≥3 | 414 (2)  164 (90-482)  280 (2)  134 (1)  17,422 (98)  304 (2)  82 (1)  28 (0.2) | 281 (1)  284 (97-690)  158 (0.4)  123 (0.3)  42,551 (99)  214 (1)  55 (0.1)  12 (0.0) | 1,496 (1)  507 (243-775)  543 (0.2)  953 (0.3)  301,751 (99.5)  1,294 (0.4)  176 (0.1)  26 (0.0) |
| **Coronary artery bypass grafting (CABG)** |  |  |  |
| CABG, n (%)  Time to first CABG, days, median (IQR)  ≤ 1 year, n (%)  > 1 year, n (%)  Number of CABG, n (%)  0  1  2  ≥3 | 74 (0.4)  129 (49-314)  59 (0.3)  15 (0.08)  17,762 (99.6)  29 (0.2)  43 (0.2)  2 (0.0) | 51 (0.1)  126 (45-467)  36 (0.1)  15 (0.05)  42,781 (99.9)  20 (0.0)  31 (0.1)  0 (0.0) | 320 (0.1)  483 (243-775)  121 (0.04)  199 (0.07)  302,927 (99.9)  107 (0.0)  208 (0.1)  5 (0.0) |
| **Emergency Department (ED) visits** |  |  |  |
| ED visits, n (%)  Time to first ED visit, days, median (IQR)  ≤ 30 days, n (%)  ≤ 1 year, n (%)  > 1 year, n (%)  Number of ER visits, n (%)  0  1  2  ≥3 | 5,454 (31)  381 (142-707) 451 (3)  2,646 (15)  2,808 (16)  12,382 (69)  3,394 (19)  1,172 (7)  888 (5) | 15,036 (35)  383 (150-688)  1,258 (3)  7,255 (18)  7,781 (18)  27,796 (65)  8,746 (20)  3,291 (8)  2,,999 (7) | 67,313 (22)  462 (215-755)  2,569 (1)  27,391 (9)  39,922 (13)  235,934 (78)  46,495 (15)  13,001 (4)  7,817 (3) |
| **General practitioner (GP), normal opening hours (8.00-16.00)** | |  |  |
| **Direct consultation** |  |  |  |
| Direct consultations, n (%)  Time to first direct consultation, days,  median (IQR)  ≤ 30 days, n (%)  ≤ 1 year, n (%)  > 1 year, n (%)  Number of direct consultations, n (%)  0  1  2  ≥3 | 17,087 (96)  31 (11-85)  8,455 (47)  16,595 (93)  492 (3)    749 (4)  464 (3)  457 (3)  16,166 (91) | 41260 (96)  31 (10-87)  20,382 (48)  39,904 (94)  1,356 (3)  1,572 (4)  1,024 (2)  1,073 (3)  39,163 (91) | 277,144 (91)  78 (28-191)  75,819 (25)  247,160 (82)  29,984 (10)  26,103 (9)  17,430 (6)  16,852 (6)  242,862 (80) |
| **ECG at first GP consultation** |  |  |  |
| ECG, n (%)  Time to first ECG, days, median (IQR)  ≤ 30 days, n (%)  ≤ 1 year, n (%)  > 1 year, n (%)  Number of ECGs, n (%)  0  1  2  ≥3 | 1,033 (6)  16 (3-86)  657 (4)  979 (6)  54 (0.3)  16,803 (94)  1,005 (6)  27 (0.2)  1 (0.0) | 3,016 (7)  13 (3-51)  2,101 (5)  2,890 (7)  126 (0.3)  39,816 (93)  2,953 (7)  62 (0.1)  1 (0.0) | 13,461 (4)  87 (30-206)  3,467 (1)  12,103 (4)  1,358 (0.5)  289,786 (96)  13,398 (4)  59 (0.0)  4 (0.0) |
| **Telephone consultations (GP)** |  |  |  |
| Telephone consultations, n (%)  Time to first telephone consultation, days, median (IQR)  ≤ 30 days, n (%)  ≤ 1 year, n (%)  > 1 year, n (%)  Number of telephone consultations, n (%)  0  1  2  ≥3 | 16,058 (90)  57 (17-168)  5,915 (33)  14,344 (80)  1,714 (10)  1,778 (10)  1,433 (8)  1,285 (7)  13,340 (75) | 38,,487 (90)  62 (17-183)  13,521 (32)  33,881 (80)  4606 (11)  4,345 (10)  3,474 (8)  3,230 (8)  31,783 (74) | 244,456 (81)  121 (38-311)  5,1445 (17)  192,281 (64)  51,075 (17)  58,791 (19)  37,564 (12)  29,459 (10)  177,433 (59) |
| **Out-of-hour consultations** |  |  |  |
| Out-of-hour consultation, n (%)  Time to first out-of-hour consultation, days, median IQR)  ≤ 30 days, n (%)  ≤ 1 year, n (%)  > 1 year, n (%)  Number of out-of-hour consultations, n (%)  0  1  2  ≥3 | 1,857 (10)  381 (155-686)  140 (1)  906 (5)  951 (5)  15,979 (90)  1,119 (6)  399 (2)  339 (2) | 7,080 (17)  345 (127-652)  681 (2)  3,697 (9)  3,183 (8)  35,752 (84)  3,999 (9)  1,499 (4)  1,582 (4) | 29,510 (10)  428 (196-723)  1,306 (0.4)  12,915 (4)  16,595 (5)  273,737 (90)  19,622 (7)  5,765 (2)  4,123 (1) |
| Death as competing risk, % | < 1.9 | < 1.0 | < 3.7 |
| Mortality, n (%) | 1,352 (8) | 2,122 (5) | 27,249 (9) |

**Table S4** Characteristics associated with healthcare contacts between women with angina or nonspecific chest pain, compared to the refence women and between the two symptomatic groups

|  | **Angina vs. reference women**  **HR (95% CI)*** | **Nonspecific chest pain vs. reference women**    **HR (95% CI)*** | **Angina vs. nonspecific chest pain**    **HR (95% CI)**** |
| --- | --- | --- | --- |
| **Readmissions** |  |  |  |
| **All cardiac readmissions, planned and unplanned** | |  |  |
| Readmission, all  Readmission, unplanned/acute  Readmission, planned | 3.24 (3.10-3.38)  2.67 (2.54-2.81)  5.41 (5.05-5.80) | 2.87 (2.78-2.97)  2.57 (2.48-2.67)  4.20 (3.96-4.45) | 1.16 (1.10-1.22)  1.07 (1.01-1.14)  1.32 (1.22-1.43) |
| **All-cause readmissions** |  |  |  |
| Readmission, all  Readmission, unplanned/acute  Readmission, planned | 1.31 (1.27-1.35)  1.26 (1.21-1.31)  1.37 (1.31-1.42) | 1.37 (1.34-1.40)  1.33 (1.30-1.37)  1.44 (1.40-1.48) | 0.98 (0.94-1.02)  0.98 (0.94-1.03)  0.96 (0.91-1.00) |
| **Procedures** |  |  |  |
| New coronary angiography (CAG)  New computed tomography angiography (CTCA)  Percutaneous Coronary Intervention (PCI)  Coronary Artery Bypass Grafting (CABG) | 8.19 (7.82-8.59)  1.47 (1.35-1.60)  4.25 (3.81-4.74)  3.39 (2.63-4.37) | 4.14 (3.96-4.33)  1.64 (1.55-1.73)  1.33 (1.17-1.51)  1.12 (0.83-1.51) | 1.96 (1.85-2.07)  0.83 (0.75-0.93)  3.29 (2.81-3.85)  3.53 (2.41-5.16) |
| **Emergency Department** |  |  |  |
| Emergency room visit | 1.41 (1.37-1.45) | 1.65 (1.62-1.68) | 0.86 (0.83-0.89) |
| **Contacts to a general practitioner (GP)** |  |  |  |
| Direct consultation  Telephone consultation  ECG  Out-of-hours consultation | 1.66 (1.63-1.69)  1.54 (1.51-1.56)  1.23 (1.16-1.31)  1.03 (0.98-1.08) | 1.68 (1.66-1.70)  1.50 (1.48-1.52)  1.68 (1.61-1.75)  1.67 (1.63-1.72) | 0.97 (0.96-0.99)  1.03 (1.01-1.05)  0.77 (0.72-0.83)  0.63 (0.60-0.67) |
|  | **Angina vs. reference women**  **IRR (95% CI)*** | **Nonspecific chest pain vs. reference women**    **IRR (95% CI)*** | **Angina vs. nonspecific chest pain**    **IRR (95% CI)**** |
| **Contacts to a general practitioner (GP)^a^** |  |  |  |
| Direct consultations  Telephone consultations | 1.57 (1.55-1.59)  1.50 (1.47-1.53) | 1.53 (1.52-1.55)  1.41 (1.39-1.42) | 1.02 (1.00-1.04)  1.07 (1.04-1.09) |
| *All analyses were adjusted for Charlson Comorbidity Index. **All analyses are adjusted for age, Body Mass Index (BMI), and Charlson Comorbidity Index. BMI was only available for symptomatic women.  ^a^ Quasi Poisson-Regression presenting the difference in the total number of events per person with results presented as incidence rate ratio (IRR) with 95% CI. | | | |

**Table S5** Characteristics associated with healthcare contacts between women with angina or nonspecific chest pain, compared to the refence women and between the two symptomatic groups

|  | **Angina vs. reference women**  **HR (95% CI)** | **Nonspecific chest pain vs. reference women**  **HR (95% CI)** | **Angina vs. nonspecific chest pain**    **HR (95% CI)** |
| --- | --- | --- | --- |
| **Readmissions** |  |  |  |
| **Cardiac readmissions** |  |  |  |
| Readmission, all*  Readmission, unplanned  Readmission, planned | 3.64 (3.48-3.79)  3.02 (2.87-3.18)  6.12 (5.72-6.56) | 2.89 (2.80-2.99)  2.61 (2.52-2.71)  4.21 (3.97-4.46) | 1.26 (1.20-1.32)  1.16 (1.09-1.22)  1.45 (1.35-1.57) |
| **All-cause readmissions** |  |  |  |
| Readmission, all*  Readmission, unplanned  Readmission, planned | 1.46 (1.42-1.50)  1.42 (1.37-1.47)  1.49 (1.43-1.55) | 1.45 (1.42-1.48)  1.42 (1.39-1.46)  1.50 (1.46-1.54) | 1.00 (0.97-1.04)  1.00 (0.96-1.04)  0.99 (0.95-1.04) |
| **Procedures** |  |  |  |
| New coronary angiography (CAG)  New computed tomography angiography (CTCA)  Percutaneous Coronary Intervention (PCI)  Coronary Artery Bypass Grafting (CABG) | 9.08 (8.67-9.51)  1.54 (1.41-1.68)  4.77 (4.28-5.32)  3.94 (3.06-5.07) | 4.15 (3.97-4.34)  1.72 (1.63-1.82)  1.33 (1.17-1.51)  1.13 (0.84-1.52) | 2.19 (2.07-2.30)  0.89 (0.81-0.99)  3.58 (3.08-4.16)  3.49 (2.44-4.99) |
| **Emergency Department** |  |  |  |
| Emergency Department visit | 1.48 (1.44-1.52) | 1.75 (1.72-1.78) | 0.84 (0.82-0.87) |
| **Contacts to a general practitioners (GP)** |  |  |  |
| Direct consultation  Telephone consultation  ECG  Out-of-hours consultation | 1.74 (1.71-1.77)  1.63 (1.61-1.66)  1.33 (1.25-1.41)  1.08 (1.44-1.52) | 1.76 (1.74-1.77)  1.57 (1.55-1.58)  1.78 (1.74-1.83)  1.78 (1.74-1.83) | 0.99 (0.97-1.00)  1.04 (1.02-1.06)  0.81 (0.76-0.87)  0.61 (0.58-0.87) |

Unadjusted analyses

*Based on unplanned/planned admissions

**Table S6** Characteristics associated with healthcare contacts using time-split Cox-regression

|  | **Angina vs. reference women**  **HR (95% CI)*** | **Nonspecific chest pain vs. reference women**    **HR (95% CI)*** | **Angina vs. nonspecific chest pain**    **HR (95% CI)**** |
| --- | --- | --- | --- |
| **Cardiac readmissions** |  |  |  |
| **Cardiac readmission, all** |  |  |  |
| Planned | 5.41 (5.05-5.80) 18.70 (16.58-21.10)^a^  3.77 (3.43-4.14)^aa^ | 4.20 (3.96-4.45) 13.88 (12.43-15.49)^a^  3.08 (2.86-3.33)^aa^ | 1.32 (1.22-1.43) |
| **Procedures** |  |  |  |
| New coronary angiography (CAG)  Percutaneous Coronary Intervention (PCI)  Coronary Artery Bypass Grafting (CABG) | 8.19 (7.82-8.59)  40.33 (37.03-43.93)^b^  4.04 (3.74-4.33)^bb^  4.25 (3.81-4.74)  14.61 (12.17-17.53)^c^  2.82 (2.43-3.29)^cc^  3.39 (2.63-4.37)  10.33 (7.29-14.66)^d^  1.60 (1.03-2.48)^dd^ | 4.14 (3.96-4.33)  20.89 (19.22-22.72)^b^  2.04 (1.90-2.18)^bb^  1.33 (1.17-1.51)  3.70 (2.98-4.61)^c^  1.07 (0.91-1.26)^cc^  1.12 (0.83-1.51)  3.32 (2.26-5.23)^d^  0.57 (0.34-0.94)^dd^ | 1.96 (1.86-2.07)  3.29 (2.81-3.85)  3.53 (2.41-5.16) |
| **Contacts to General Practitioners** |  |  |  |
| Electrocardiogram (ECG) | 1.23 (1.16-1.31)  3.22 (2.96-3.50)^e^  0.67 (0.60-0.74)^ee^ | 1.68 (1.61-1.75)  4.70 (4.45-4.96)^e^  0.76 (0.71-0.81)^ee^ | 0.77 (0.72-0.83) |
| *All analyses were adjusted for age, and the Charlson Comorbidity Index; **All analyses were adjusted for age, the Charlson Comorbidity Index, and the Body Mass Index (BMI). BMI was only available for the angina and nonspecific chest pain group.  ^a^ The association to the outcome until 183 days after the first event ^aa^The association to the outcome after 183 days and until the end of follow-up time.  ^b^ The association to the outcome until 180 days after the first event ^bb^The association to the outcome after 180 days and until the end of follow-up time.  ^c^ The association to the outcome until 183 days after the first event ^cc^The association to the outcome after 183 days and until the end of follow-up time.  ^d^ The association to the outcome until 270 days after the first event ^dd^ The association to the outcome after 270 days and until the end of follow-up time.  ^e^ The association to the outcome until 180 days after the first event ^ee^ The association to the outcome after 180 days and until the end of follow-up time. | | | |

**Table S7** The association between angiographic procedure (CAG vs CTCA) and long-term prognostic outcomes (2009-2022) in women with angina or nonspecific chest pain.

|  | **Women with symptoms of angina**  **HR (95% CI)*** | | **Women with Nonspecific chest pain**  **HR (95% CI)*** | |
| --- | --- | --- | --- | --- |
|  | **Mortality** | | **Mortality** | |
|  | Unadjusted (crude) | Adjusted | Unadjusted (crude) | Adjusted |
| CAG vs CTCA (ref) | 2.84 (2.48-3.24) | 1.65 (1.43-1.91) | 2.95 (2.75-3.18) | 1.73 (1.60-1.87) |
|  | **ACS** | | **ACS** | |
|  | Unadjusted (crude) | Adjusted | Unadjusted (crude) | Adjusted |
| CAG vs CTCA (ref) | 2.42 (1.94-3.02) | 2.33 (1.85-2.94) | 2.97 (2.59-3.39) | 2.95 (2.56-3.39) |
|  | **Heart failure** | | **Heart failure** | |
|  | Unadjusted (crude) | Adjusted | Unadjusted (crude) | Adjusted |
| CAG vs CTCA (ref) | 1.97 (1.55-2.51) | 1.68 (1.30-2.16) | 2.16 (1.90-2.46) | 1.83 (1.60-2.09) |

*Adjusted for age, Charlson Comorbidity Index (CCI), Body Mass Index (BMI). Patient who underwent both CTCA and CAG were included in the CAG group.

**Figure S1a, b, and c.** Accumulated new events from the initial diagnosis up to three years of follow-up divided into cardiac readmission, planned, all-cause readmission, unplanned, all-cause readmission, planned (S1a), new CTCA, PCI, CABG (S1b), and GP, direct consultation, GP, telephone consultation, ECG at first GP consultation (S1c).

Cumulative incidence function with a 95% confidence interval of the cumulative events within three years of follow-up. All survival curves include confidence intervals.

**Figure S2** Barchart demonstrating the proportion of women experiencing the first event within one month, one year, and after one year

The y-axis is the proportions of healthcare contacts among reference women and women with angina/nonspecific chest pain with a maximum of 100%.
