# Supplementary figures and images for: The use of healthcare contacts following a first diagnosis of chest pain among women with no obstructive coronary artery disease: results from the WOMANOCA nationwide cohort study
Source: Eur Heart J Qual Care Clin Outcomes. 2025 Jun 27;11(8):1396–408. doi: 10.1093/ehjqcco/qcaf051 (PMC12714392; doi:10.1093/ehjqcco/qcaf051)

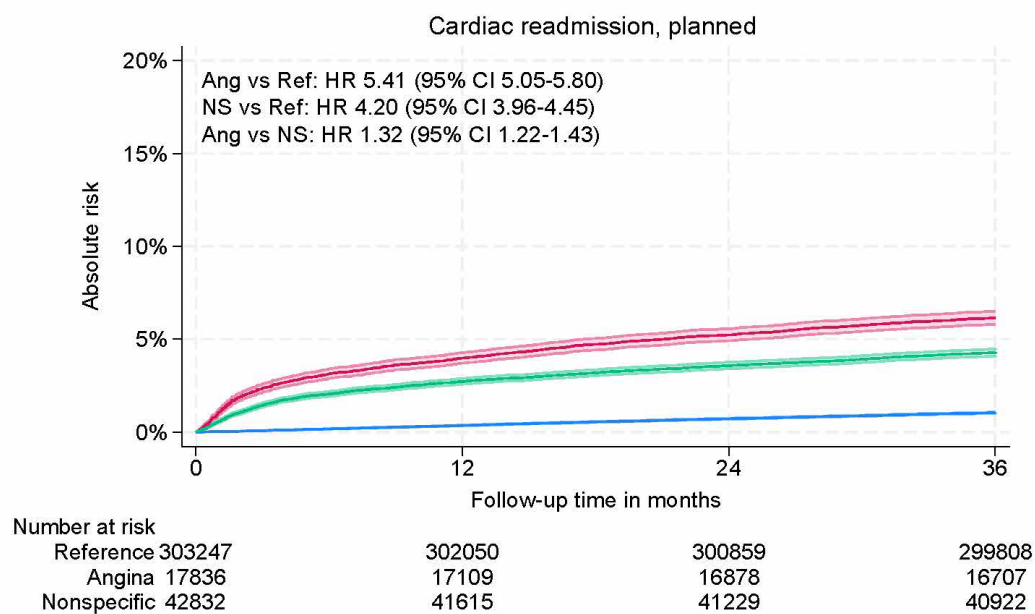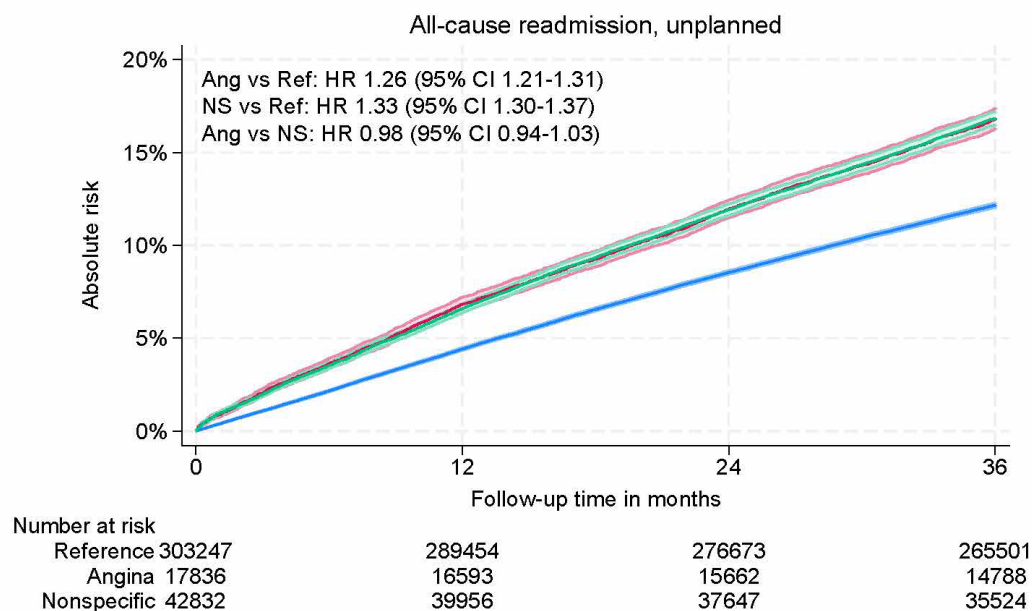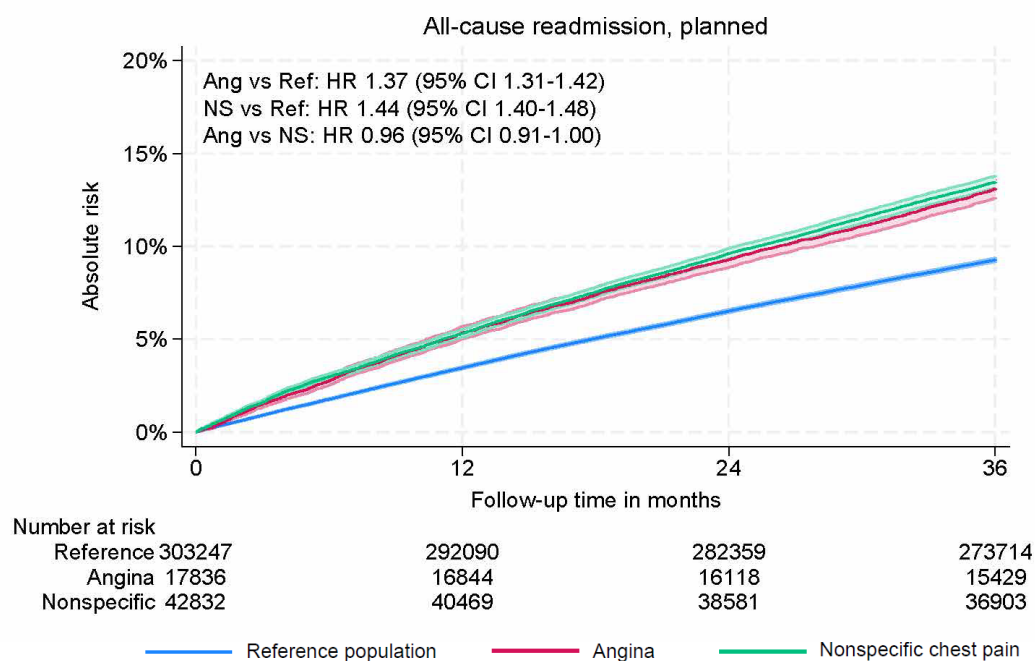

Supplement: qcaf051_Supplementary_Data [file qcaf051_supplementary_data.zip › FigS1a_JLDA.pdf]

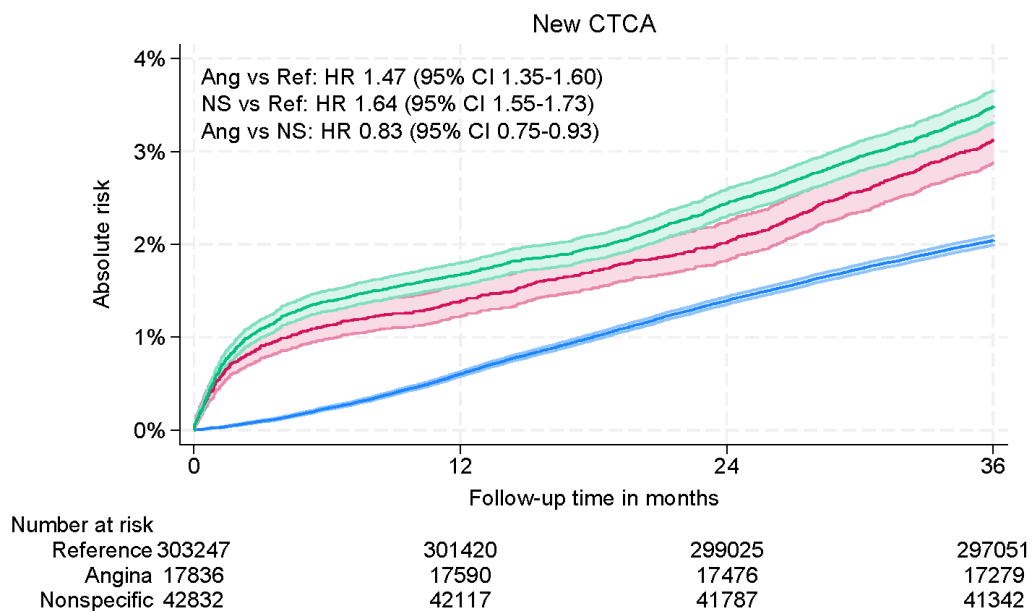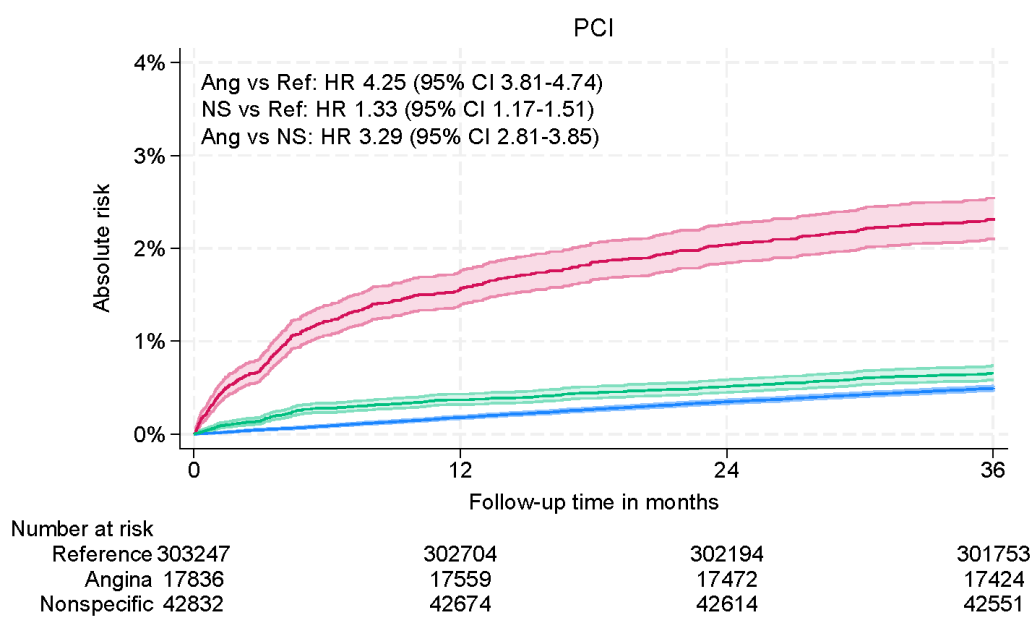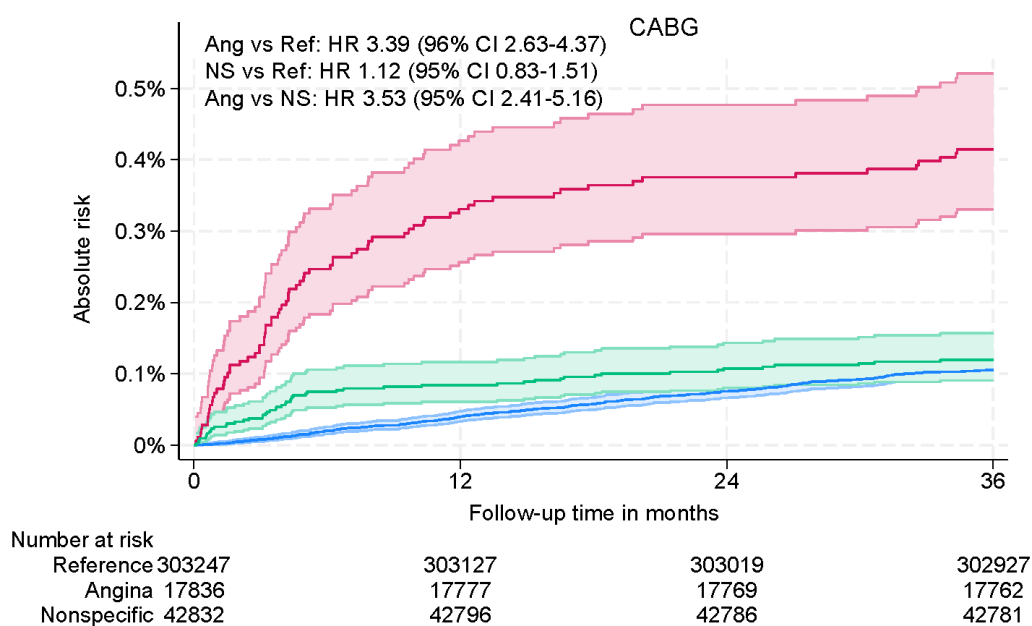

— Reference population      — Angina      — Nonspecific chest pain

Supplement: qcaf051_Supplementary_Data [file qcaf051_supplementary_data.zip › FigS1b_JLDA.pdf]

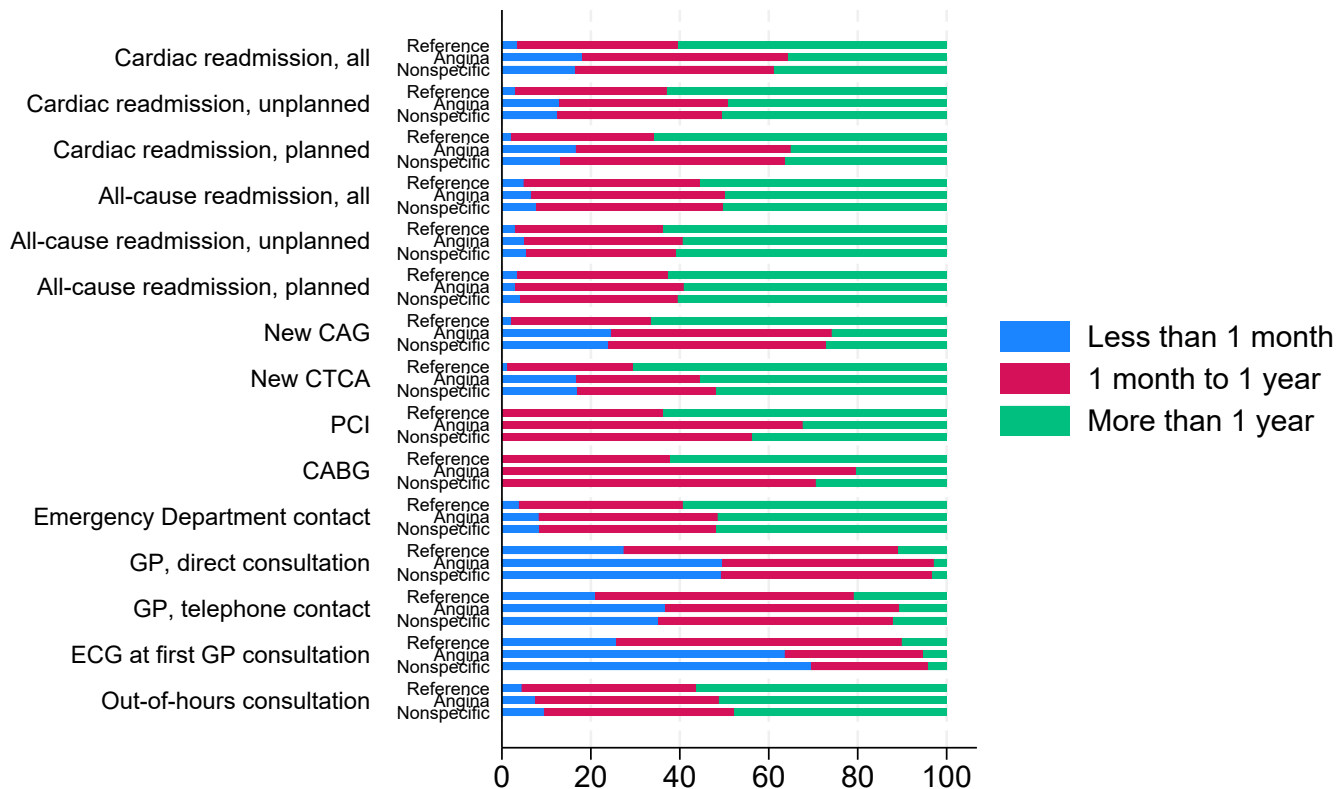

Supplement: qcaf051_Supplementary_Data [file qcaf051_supplementary_data.zip › FigS2_JLDA.pdf]
